# Flavonoids Targeting HIF-1: Implications on Cancer Metabolism

**DOI:** 10.3390/cancers13010130

**Published:** 2021-01-03

**Authors:** Marek Samec, Alena Liskova, Lenka Koklesova, Sandra Mersakova, Jan Strnadel, Karol Kajo, Martin Pec, Kevin Zhai, Karel Smejkal, Sepideh Mirzaei, Kiavash Hushmandi, Milad Ashrafizadeh, Luciano Saso, Aranka Brockmueller, Mehdi Shakibaei, Dietrich Büsselberg, Peter Kubatka

**Affiliations:** 1 Clinic of Obstetrics and Gynecology, Jessenius Faculty of Medicine, Comenius University in Bratislava, 03601 Martin, Slovakia; marek.samec@uniba.sk (M.S.); liskova80@uniba.sk (A.L.); koklesova.lenka@gmail.com (L.K.); 2 Biomedical Centre Martin, Jessenius Faculty of Medicine in Martin, Comenius University in Bratislava, Mala Hora 4D, 03601 Martin, Slovakia; mersakova1@uniba.sk (S.M.); jan.strnadel@uniba.sk (J.S.); 3 Department of Pathology, St. Elizabeth Cancer Institute Hospital, 81250 Bratislava, Slovakia; kkajo@ousa.sk; 4 Department of Medical Biology, Jessenius Faculty of Medicine, Comenius University in Bratislava, 03601 Martin, Slovakia; martin.pec@uniba.sk; 5 Department of Physiology and Biophysics, Weill Cornell Medicine in Qatar, Education City, Qatar Foundation, Doha 24144, Qatar; kez4003@qatar-med.cornell.edu; 6 Department of Natural Drugs, Faculty of Pharmacy, Masaryk University, Palackého třída 1946/1, 61200 Brno, Czech Republic; karel.mejkal@post.cz; 7 Department of Biology, Faculty of Science, Islamic Azad University, Science and Research Branch, 1477893855 Tehran, Iran; sepidehmirzaei.smv@gmail.com; 8 Department of Food Hygiene and Quality Control, Division of Epidemiology, Faculty of Veterinary Medicine, University of Tehran, 1419963114 Tehran, Iran; houshmandi.kia7@ut.ac.ir; 9 Faculty of Engineering and Natural Sciences, Sabanci University, Orta Mahalle, Üniversite Caddesi No. 27, Orhanlı, Tuzla, 34956 Istanbul, Turkey; milad.ashrafizadeh@sabanciuniv.edu; 10 Sabanci University Nanotechnology Research and Application Center (SUNUM), Tuzla, 34956 Istanbul, Turkey; 11 Department of Physiology and Pharmacology “Vittorio Erspamer”, Faculty of Pharmacy and Medicine, Sapienza University, 00185 Rome, Italy; luciano.saso@uniroma1.it; 12 Musculoskeletal Research Group and Tumor Biology, Chair of Vegetative Anatomy, Institute of Anatomy, Faculty of Medicine, Ludwig-Maximilian-University Munich, D-80336 Munich, Germany; Aranka.Brockmueller@med.uni-muenchen.de (A.B.); mehdi.shakibaei@med.uni-muenchen.de (M.S.)

**Keywords:** cancer, Warburg effect, HIF-1, flavonoids

## Abstract

**Simple Summary:**

This comprehensive review discusses the anticancer effects of plant phenolic compounds, known as flavonoids, through the targeting of HIF-1 and critical enzymes contributing to the Warburg effect. Connections between HIF-1 and metabolic reprogramming seem to play a crucial role in cancer progression. The core of presented paper summarizes the current knowledge about the in vitro and in vivo efficacy of flavonoids against aerobic glycolysis and HIF-1 activity. Despite the lack of clinical evidence, we emphasize the possibility of introducing flavonoids (targeting HIF-1) to the clinical research considering predictive, preventive, and/or personalized medical approach.

**Abstract:**

Tumor hypoxia is described as an oxygen deprivation in malignant tissue. The hypoxic condition is a consequence of an imbalance between rapidly proliferating cells and a vascularization that leads to lower oxygen levels in tumors. Hypoxia-inducible factor 1 (HIF-1) is an essential transcription factor contributing to the regulation of hypoxia-associated genes. Some of these genes modulate molecular cascades associated with the Warburg effect and its accompanying pathways and, therefore, represent promising targets for cancer treatment. Current progress in the development of therapeutic approaches brings several promising inhibitors of HIF-1. Flavonoids, widely occurring in various plants, exert a broad spectrum of beneficial effects on human health, and are potentially powerful therapeutic tools against cancer. Recent evidences identified numerous natural flavonoids and their derivatives as inhibitors of HIF-1, associated with the regulation of critical glycolytic components in cancer cells, including pyruvate kinase M2(PKM2), lactate dehydrogenase (LDHA), glucose transporters (GLUTs), hexokinase II (HKII), phosphofructokinase-1 (PFK-1), and pyruvate dehydrogenase kinase (PDK). Here, we discuss the results of most recent studies evaluating the impact of flavonoids on HIF-1 accompanied by the regulation of critical enzymes contributing to the Warburg phenotype. Besides, flavonoid effects on glucose metabolism via regulation of HIF-1 activity represent a promising avenue in cancer-related research. At the same time, only more-in depth investigations can further elucidate the mechanistic and clinical connections between HIF-1 and cancer metabolism.

## 1. Introduction

Despite amazing progress in understanding, diagnosis, therapy, and prevention, cancer remains one of the leading causes of death worldwide, with more than 18 million new cases detected in 2018 [1,2]. Cancer is characterized as a multistage process in which tumor cells acquire specific abilities, such as uncontrolled proliferation, avoidance of apoptosis, invasiveness, and the promotion of neovascularization [3]. The uncontrolled and rapid proliferation of tumor cells and insufficient formation of new blood vessels lead to inadequate oxygen supply to tumor tissues. Therefore, it is not unusual for developing malignant tissue to possess hypoxic and necrotic areas [4]. A cell’s adaptation to a lower level of oxygen in hypoxic regions is accompanied by the activation of several survival pathways [5]. Hypoxia-inducible factor 1 (HIF-1) is a crucial transcription factor responsible for the regulation of hypoxic responses. Recent evidence has revealed that an elevated level of HIF-1 could act as a prognostic marker associated with metastasis, angiogenesis, development of chemo/radioresistance, and overall poor prognosis of cancer patients [6,7]. Metabolic reprogramming, leading to the switch from oxidative phosphorylation (OXPHOS) to aerobic glycolysis, is crucial for cell adaptation to a hypoxic environment. The favoring of aerobic glycolysis over OXPHOS (known as the Warburg effect), even at normal oxygen levels, is frequently observed in many solid tumors [8].

The stabilization of HIF-1 independently of hypoxia could explain glycolysis’s acceleration under normoxic conditions. Experimental studies have recently suggested a critical role of HIF-1 in regulating critical glycolytic proteins that contribute to the Warburg phenotype; thus, HIF-1 represents a potential target against metabolic reprogramming in cancer cells [9]. Due to its significant cancer development role, several HIF-1 inhibitors were recently clinically trailed. However, further studies on HIF-1 are necessary to provide novel therapeutic tools to inhibit its activity. Flavonoids, a class of naturally occurring plant-derived compounds, exert numerous beneficial human health attributes [10]. Flavonoids modulate various signaling pathways associated with cancer initiation, promotion, and progression, both in vitro and in vivo [4,11,12,13]. The effects of flavonoids on the regulatory cascade connected to HIF-1 and glucose metabolism constitute a promising way to inhibit metabolic reprogramming via the regulation of HIF-1 activity, as well as critical components of glycolysis. Therefore, in this review, we provide a comprehensive discussion of recent studies evaluating the inhibitory effects of flavonoids on HIF-1 and proteins directly contributing to the Warburg effect. The anticancer effectiveness of dietary phenols supports their application in preclinical as well as clinical research, but several complications associated with bioavailability and safety must be overcome to eliminate flavonoids’ side effects. Although flavonoids, either independently or combined with conventional therapies, could act as powerful therapeutic tools targeting cancer, further mechanistic evaluation and identifying individuals who would benefit from flavonoid-based approaches can provide hope for cancer patients.

### 1.1. Aim of the Study

This comprehensive review discusses the anticancer effects of plant phenolic compounds, known as flavonoids, through the targeting of HIF-1 and critical enzymes contributing to the Warburg effect. Connections between HIF-1 and metabolic reprogramming play a crucial role in cancer progression. The core of presented paper summarizes the current knowledge about the in vitro and in vivo efficacy of flavonoids against aerobic glycolysis and HIF-1 activity. Despite the lack of clinical evidence, we emphasize the possibility of introducing flavonoids (targeting HIF-1) to the clinical research considering predictive, preventive, and/or personalized medicine.

### 1.2. Source of the Data

The presented data were obtained from biomedical literature through the use of “hypoxia” and “HIF-1” or “ Warburg effect” or “aerobic glycolysis” or “flavonoids” or “flavonols” or “chalcones” or “anthocyanidins” or “flavanols” or “flavones” or “isoflavonoids” or “flavanones” as either keywords or medical subject heading (MeSH) terms in searches of the PubMed database. We focused on recent publications from the last five years (2016–2020).

## 2. Hypoxic Conditions

Hypoxia is defined as oxygen deficiency that results in inadequate tissue oxygenation. A low oxygen level is a characteristic feature of cancer tissue. Rapid tumor growth leads to a reduced oxygen supply to specific cancer tissue areas due to insufficient vasculature development [9]. Beyond hypoxia’s role in the neovascularization necessary for the adaptation of cancer cells to oxygen and nutrient deprivation, the hypoxic state is related to other tumor features such as metabolic alterations, prolonged cell lifespan, and changes in cell adhesion and production of the extracellular matrix [14,15]. Therefore, hypoxia is a hallmark of solid tumors and is strongly connected with poor clinical prognosis due to the development of chemoresistance, radioresistance, or more aggressive forms of the disease resulting in metastasis [16,17]. The mechanism of tumor adaptation to hypoxic conditions is mediated by hypoxia-inducible factors (HIFs), which are transcription factors targeting specific genes in response to low oxygen levels [18].

### 2.1. Structure of Hypoxia-Inducible Factor 1

**Figure 1:**
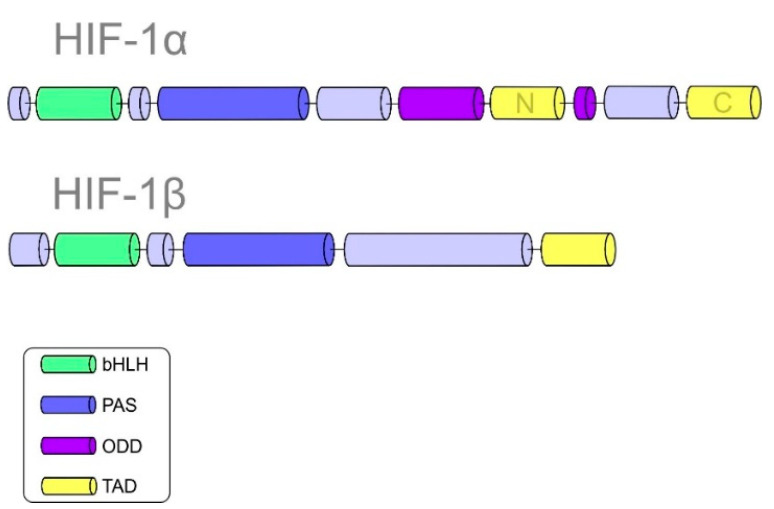
Domain structures of hypoxia-inducible factor 1 (HIF-1α) and HIF-1β.

HIF-1 plays an essential role in cellular adaptation to hypoxia. Structurally, HIF-1 is a heterodimeric protein composed of HIF-α and HIF-β subunits [19]. Subunit α is further divided into three isoforms: HIF-1α, HIF-2α, and HIF-3α [20]. It is important to note that HIF-β is a constitutively expressed subunit, while HIF-α is an oxygen-regulated component of HIF-1. The helix-loop-helix domain (bHLH), responsible for DNA binding, allows the dimerization of both α and β subunits. HIF-1α and HIF-1β are composed of Per-ARNT-Sim (PAS) domains that are essential for maintaining the heterodimerization of the α and β subunits [21]. The α subunit is characterized by the oxygen-dependent degradation (ODD) domain. Prolyl-hydroxylase-2 (PHD-2) hydroxylates the ODD domain, resulting in the proteasomal degradation of the α subunit under normoxic conditions [22,23]. Other significant domains of HIF-1α include the N-terminal transactivational domain (N-TAD), which is essential for the activation of HIF-1 target genes, and the C-terminal domain (C-TAD). The C-TAD domain interacts with the C-TAD binding protein (CBP) and P300 to regulate the transcription of HIF-1α under hypoxic conditions [5,24]. Figure 1 summarizes the structures of both HIF-1α and HIF-1β subunits with their specific domains.

### 2.2. Regulation of HIF-1

As mentioned above, HIF-1β is a constitutively expressed subunit, while different pathways can regulate the expression of HIF-1α. HIF-1α is degraded via the ubiquitin-proteasome pathway under normoxic conditions. Prolyl hydroxylases (PHDs) have an essential role in hydroxylating HIF-1α at its proline residues in an oxygen-dependent manner [25]. The hydroxylation of proline residues localized on the α subunit leads to ubiquitination mediated by von Hippel-Lindau suppressor (pVHL), which contains E3 ubiquitin ligase, and subsequent degradation of HIF-1α in the proteasome [26]. Importantly, PHD is inhibited, and HIF-1α is therefore stabilized under hypoxic conditions. The second way of oxygen-dependent regulation of HIF-1α is a cooperation with factor inhibiting HIF1 (FIH). FIH affects the stability of HIF-1α via hydroxylation of its asparagine residues. This interaction causes the inhibition of transcriptional coactivator recruitment [27]. Moreover, FIH and PHD are dependent on the intracellular oxygen concentration, and both require cooperation with co-factors, including 2OG (α-ketoglutarate) and Fe^2+^ ions. In the absence of the mentioned co-factors, HIF-1α is active even under normoxic conditions [28]. Interestingly, calcium-mediated regulation of HIF-1α is another way to modulate the activity of transcription factors. Recent evidence shows a strong correlation between disequilibrium in intracellular calcium homeostasis and HIF-1 activity [29]. For instance, calcium affects the dimerization of the receptor of activated protein C kinase (RACK1), resulting in the regulation of HIF-1α [30]. Additionally, the oxygen-independent manner of HIF-1 regulation is associated with various signaling pathways [31]. HIF-1α is affected by the ERK/MAPK [32], PI3K/AKT/mTOR [33], and JAK/STAT [29] signaling cascades. E3 ubiquitin ligase Mdm2 has an essential role in the regulation of transcription factors, including HIF-1α and p53. The interplay between p53 and HIF-1α was documented in normoxia when p53 binds to HIF-1α, leading to proteasomal degradation via Mdm2. Loss of or mutations in the tumor suppressor p53 inhibit Mdm2-mediated proteasomal degradation of HIF-1α [34]. Besides, heat shock chaperone protein 90 (HSP-90) is responsible for the stabilization of HIF-1α via conformational changes in its structure [35]. Furthermore, many studies described an important role of epigenetic mechanisms in the regulation of HIF-1 stability and activity [36,37,38]. Enzymes associated with epigenetic machinery in cells include methyltransferases, acetyltransferases, ubiquitin E3 ligases, and protein kinases that act as epigenetic writers. These enzymes can add epigenetic marks onto RNA, DNA, or histones [36,39]. Histone acetyltransferases, including c300/CBP and p300/CBP-associated factor (PCAF), promote HIF-1α stability. On the other hand, acetyltransferase arrest-defective-1 (ARD1) induces the acetylation of lysine residues and consequent ubiquitination of the HIF-1α subunit [40]. In the case of methyltransferases, methyltransferase SET7/9 is a regulator of HIF-1α stability [38]. Moreover, the HIF-α regulatory capacity of methyltransferases (PRMT1, PRMT5, and PRMT9) was detected at different levels [36,41,42]. Epigenetic erasers (demethylases and deacetylases) are enzymes responsible for removing specific epigenetic marks (such as methyl groups from chromatin) and, similarly to epigenetic writers, participate in chromatin remodeling and gene regulation. For instance, histone deacetylases (HDACs) (HDAC1, -2, -3, -4, -6) increase stability of HIF-1α protein [43,44,45,46]. Furthermore, HDAC7 interacts with HIF-1α and p300/CBP, and thereby promotes HIF-1 transcriptional activity. Furthermore, lysine-specific demethylase 1 (LSD1) promotes the increased stability of HIF-1α by suppressing RACK1-mediated HIF-1α degradation [38]. DNA methylation also modulates HIF-1α stability and activity. Hypermethylation of the VHL promoter region leads to constitutive activation of HIF-1α [47,48]. Numerous studies have observed a connection between HIF and non-coding RNAs. Hypoxia can enhance the expression of non-coding RNAs that can modulate HIF expression and stability. MicroRNAs, a group of small non-coding RNAs, regulate the expression of target genes at the post-transcriptional level [49]. Recent evidence suggests interplay between HIF-1α and miR-33b, miR-338-3p, miR-138, miR-576-3p, miR-143-3p, and miR-20b [50,51,52,53,54,55]. Interestingly, the regulatory role of long non-coding RNA (lncRNA) was detected in gallbladder cancer. The lncRNA LINC00152 was identified as an oncogene due to its role as a miR sponge for miR-138 targeting HIF-1α to promote carcinogenesis [56]. Similarly, the oncogenic character of lncRNAs acting as competing endogenous RNAs (ceRNAs) associated with HIF-1α was documented by HOX transcript antisense RNA (HOTAIR) (suppression of miR-217), nuclear paraspeckle assembly transcript 1 (NEAT1) (suppression of miR-186-5p), and PVT1 (suppression of miR-186) [57,58,59].

### 2.3. A Brief Introduction to the Warburg Phenotype

Alterations in tumor cell metabolism are a fundamental aspect of cancer compared to non-malignant cells [20]. In healthy cells, glucose is metabolized to pyruvate through the glycolytic cascade and subsequently oxidized to CO_2_ in mitochondria via oxidative phosphorylation. This metabolic cascade generates 36 molecules of ATP per molecule of glucose. The described pathway occurs under the normoxic condition of healthy cells [60]. During hypoxia, mitochondrial OXPHOS is restricted, leading to pyruvate conversion into lactate in a process called aerobic glycolysis [61]; notably, the overall yield of aerobic glycolysis is only 2 ATP per molecule of glucose.

Interestingly, the preference for aerobic glycolysis even under normoxic conditions and with fully functioning mitochondria is observed across different cancer types [62]. This phenomenon of metabolic reprogramming in cancer cells was first described almost 100 years ago by Otto Warburg [62]. The observation revealed that cancer cells receive enormous amounts of glucose compared to surrounding non-malignant tissue. The repression of cancer cell respiration and the enhancement of glucose fermentation into lactate were also identified [11,63]. Recent evidence has revealed the specific molecular mechanisms responsible for switching from OXPHOS to aerobic glycolysis. Alterations in molecular pathways, including PI3K/Akt/mTOR, specific genes such as *cMYC* and *p53*, and changes in epigenetic machinery directly participate in the regulation of cancer metabolism [11,64,65,66,67]. HIF-1 plays an essential role in the regulation of changes leading to the Warburg phenotype; it acts as a central regulator of glucose metabolism and cell proliferation.

### 2.4. Implementation of HIF-1 in the Modulation of Cancer Metabolism

**Figure 2:**
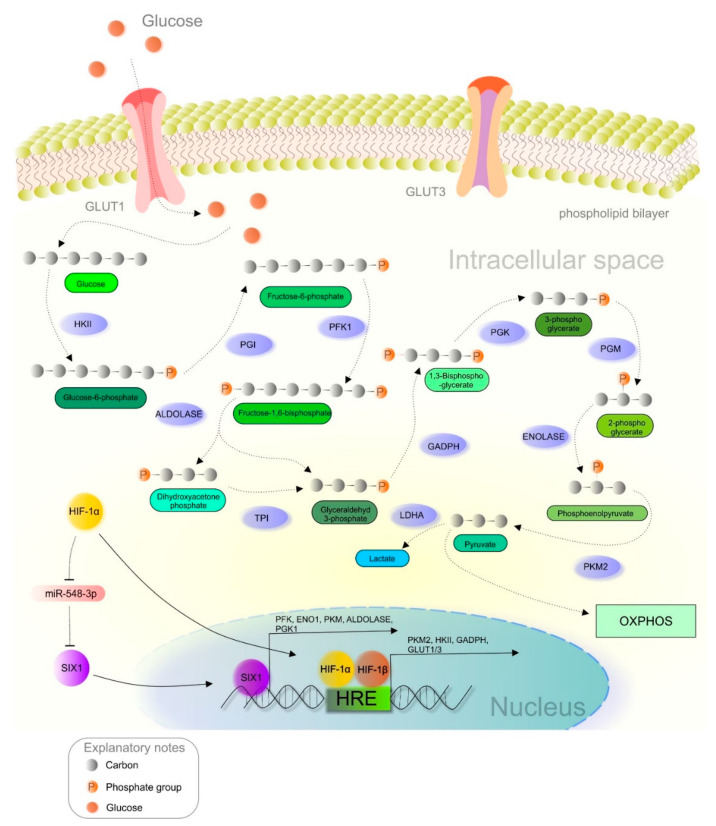
HIF-1α-mediated crosstalk between hypoxia and glucose metabolism in a cancer cell. Abbreviations: HKII, hexokinase II; PGI, phosphoglucose isomerase; PFK1, phosphofructokinase; TPI, triosephosphate isomerase; GAPDH, glyceraldehyde-3-phosphate dehydrogenase; PGK, phosphoglycerate kinase; PGM, phosphoglycerate mutase; PKM2, pyruvate kinase M2; LDH, lactate dehydrogenase; ENO1, enolase 1; OXPHOS, oxidative phosphorylation; HRE, hypoxia-response elements; HIF-1α/1β, hypoxia-inducible factor 1α/1β; GLUT1/3, glucose transporter 1/3.

As principally described in the previous section, the metabolic reprogramming of cancer cells allows the switch from OXPHOS to less efficient aerobic glycolysis, regulated by various molecular events such as the HIF-1 regulatory pathway (Figure 2) [11,68]. Dimerization of both HIF subunits (α and β) initiates the transcription of genes associated with increased glucose uptake and lactate production. Heterodimer HIF-1 binds to the hypoxia-response element (HRE) and begins expressing hypoxia-responsive genes [8,69]. Glucose transporters (GLUTs) are responsible for transporting glucose into the cells, and hypoxia leads to the upregulation of GLUT1 and GLUT3 [70]. Overexpression of GLUTs guarantees enough glucose for the cancer cells. Correlations between elevated HIF-1 activity and the overexpression of GLUT1 and GLUT3 were observed in glioma [71] and pancreatic neuroendocrine tumors, respectively [72]. Pyruvate kinase (PK), an enzyme responsible for converting phosphoenolpyruvate (PEP) into pyruvate, comprises four isoforms (PKL, PKR, PKM1, and PKM2). Notably, the PKM2 isoform is preferred in cancer cells, while PKM2 gene transcription is modulated by HIF-1 [73]. PKM2 acts as a coactivator via interaction with the HIF-1α subunit due to oxygen deprivation or oncogene activation [74]. This interaction promotes the transactivation of target genes regulated by HIF-1 [75]. Hexokinase (HK), another glycolytic enzyme associated with enhanced glucose metabolism, is often upregulated in poorly differentiated and highly proliferated neoplastic tissues [76]. Recently, the connections between aberrantly triggered nuclear factor-κB (NF-κB), HIF-1, and hexokinase II (HKII) expression were observed in B-cell lymphoma, while NF-kB inhibitors suppressed HIF-1 and HKII [77]. Similarly, glyceraldehyde-3-phosphate dehydrogenase (GAPDH) is an enzyme that catalyzes the conversion of glyceraldehyde-3-phosphate to 1,3-biphosphoglycerate. Notably, this enzyme is often upregulated in cancer, as demonstrated in colorectal [78] and colon tumors, [79] and melanoma [80]. Deeper analysis of GADPH also revealed an association between the enzyme’s activity and HIF-1 expression in breast cancer [81]. Interestingly, the transcription of genes associated with the Warburg phenotype, including *GLUT1*, phosphofructokinase (*PFK*), enolase-1 (*ENO1*), *PKM*, aldolase A (*ALDOA*), and phosphoglycerate kinase-1 (*PGK1*), is regulated by the SIX1 transcription factor through cooperation with the histone acetyltransferases HBO1 and AIB1. Current evidence describes an essential role of miR-548a via the suppression of SIX1. Additionally, elevated HIF-1α levels are associated with decreased miR-548a, and thus enhance glycolysis and tumor growth [82].

### 2.5. Therapeutic Interventions Based on HIF-1 Regulation: Current Status and Future Directions

As noted above, the HIF-1 pathway modulates the expression of numerous genes involved in cancer progression and the development of resistance to different treatments [83]. Therefore, HIF, particularly its subunits HIF-1α and HIF-2α, represents a potential target of novel and potentially clinically viable oncologic interventions [84]. Several HIF inhibitors were documented to target cancer in preclinical and clinical trials. Based on their mechanisms of HIF repression, therapeutic agents are classified as factors regulating gene expression, factors modulating protein synthesis, agents modulating cascades associated with protein dimerization and accumulation, or inhibitors responsible for DNA binding and the transcriptional activity of HIF-1.

EZN-2968, an antisense oligonucleotide targeting HIF-1α mRNA, modulates HIF-1 activity at a transcriptional level; it was clinically trailed (NCT01120288, NCT02564614) [85]. Similarly, topotecan (a topoisomerase 1 inhibitor) demonstrated activity against the hypoxic phenotype by inhibiting HIF-1α mRNA expression [86,87]. Inhibitors of HIF-1α and HIF-2α protein synthesis include 2-methoxyestradiol, which affects protein synthesis in lung cancer cells [88], and KC7F2, which markedly suppresses the synthesis of HIF-1α in glioma, prostate, and breast cancers [89,90]. Another possible way to modulate HIF-1 activity is the disruption of protein stabilization and accumulation. Hsp90 inhibitors, such as geldanamycin, tanespimycin, and alvespimycin, degrade HIF-1α through a VHL-independent proteasomal mechanism [91,92,93]. Moreover, histone deacetylase inhibitors (HDACi), such as vorinostat exerted anti-hypoxic activity by degrading the HIF-1α subunit in liver cancer-derived cells [94]. Disruption of HIF-1α dimerization represents a promising way to inhibit its transcriptional activity. Recent evidence revealed an essential role of a cyclic peptide (cyclo-CLLFVY) via inhibition of HIF-1α heterodimerization [19,95].

Acriflavine showed potential as a therapeutic tool that disrupts HIF-1α dimerization [96]. Interestingly, doxorubicin and daunorubicin, well known chemotherapeutic agents, also exerted hypoxia-modulating abilities by inhibiting the binding of HIF-1 to its target (HRE) sequences [97]. Finally, epidithiodiketopiperazine chetomin inhibited the transcriptional activity of HIF-1 by targeting the HIF-1α/p300 complex in multiple myeloma cell lines [98].

Table 1 provides an overview of inhibitors that modulate HIF-1 activity at different levels of action. As was briefly analyzed in this section, inhibitors of HIF-1 are widely tested in preclinical and clinical research. Their multilevel mechanisms of action show novel opportunities in therapy and open a hidden chamber of hypoxia’s molecular secrets for a more profound understanding of the hypoxia-associated cascade in cancer. Further research in this sphere can bring well-deserved rewards in the form of effective therapeutic interventions without side effects for cancer patients.

## 3. Beneficial Effects of Flavonoids in the Regulation of Hypoxic Molecular Cascades and the Warburg Effect

**Figure 3:**
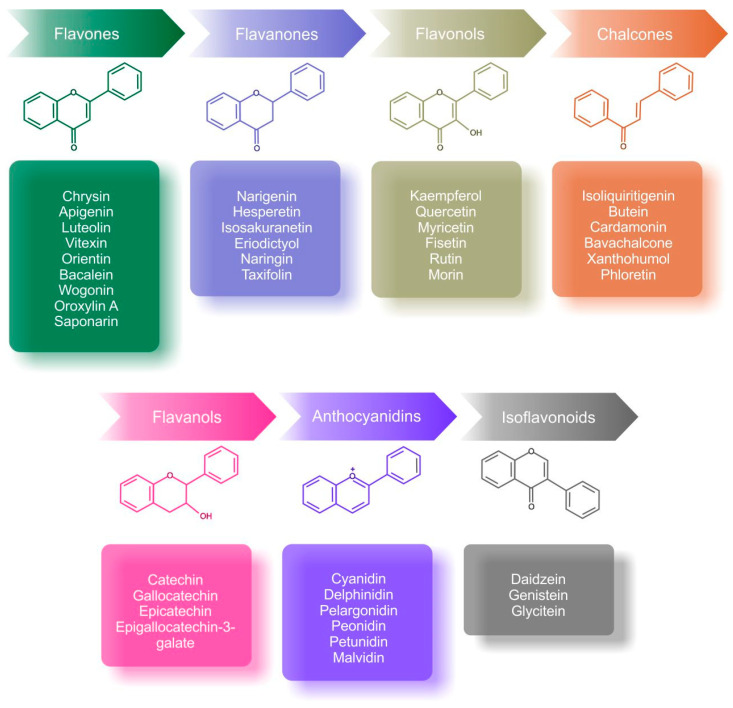
Classification and chemical structures of the main subgroups of flavonoids with examples [99,114,115,116,117].

Flavonoids are secondary metabolites of plants with specific phenolic structures. These bioactive compounds are found in numerous fruits, vegetables, roots, bark, flowers of medicinal plants, and in beverages such as wine and tea [10,11,99]. Chemically, flavonoids are composed of a fifteen-carbon skeleton consisting of benzene rings (A and B) and a heterocyclic pyran ring (C) [100,101]. Flavonoids are classified into seven structural classes: flavanones, flavonols, chalcones, flavanols, anthocyanidins, flavones, and isoflavonoids (Figure 3) [12]. As dietary components, flavonoids have numerous beneficial properties for human health, including anti-oxidant [102], anti-inflammatory [103], anti-bacterial [104], anti-fungal [105], and anti-viral activities [106]. Recent evidence suggests that dietary phenols like flavonoids support the inhibition of cancer initiation, promotion, and progression both in vitro and in vivo [107]. Moreover, flavonoids exert anti-angiogenic [13] and anti-proliferative [108] effects and can modulate epigenetic mechanisms associated with carcinogenesis [109].

As discussed above, hypoxia is directly connected with various neoplastic processes. HIF-1 represents a critical transcription factor that is closely related to the regulation of genes responsible for angiogenesis [110], metastasis [111], cell survival, proliferation [112], and apoptosis [113]. Moreover, changes in cancer metabolism, which lead to the Warburg phenotype and represent a switch from OXPHOS to aerobic glycolysis, are also regulated by HIF-1. The regulation of enzymes involved in glucose metabolism, including HK, PK, PFK, lactate dehydrogenase (LDHA), pyruvate dehydrogenase kinase (PDK) 1, and GLUTs, is influenced by HIF-1 activity [8]. Thus, HIF-1 represents a promising target for clinical interventions reducing cancer occurrence and suppressing tumor development; these interventions may regulate its expression, modulate protein synthesis, affect protein dimerization, or decrease the ability of HIF-1 to bind to its target DNA sequences. The promising effects of flavonoids in the modulation of hypoxia-associated cascades represent a unique opportunity to inhibit pathways leading to aerobic glycolysis in cancer cells.

### 3.1. Regulation of HIF-1 Activity by Flavonoids

The anticancer activities of flavonoids are mediated in part via the inhibition of HIF-1. HIF-1 is a critical transcription factor targeting essential genes contributing to the Warburg phenotype. Therefore, it is necessary to comprehensively discuss the effects of secondary plant metabolites on HIF-1 activity [4]. In this regard, we summarize the current status of experimental studies that focus on the modulation of HIF-1 activity by flavonoids in cancer models.

Quercetin is a commonly occurring flavonol with anti-inflammatory and antioxidant properties. Quercetin exerts promising anti-tumor effects via the regulation of various cancer signaling pathways [118], including effect on activity of HIF-1. Quercetin inhibited HIF-1 transcriptional activity in the HCT116 colon cancer cell line. Besides, the reduction of HIF-1 activity was related to the inhibition of AMP-activated protein kinase (AMPK) activity, resulting in quercetin-induced apoptosis under hypoxic conditions in vitro. Moreover, the administration of quercetin in vivo attenuated tumor growth in a xenograft model [119]. Similarly, quercetin inhibited HIF-1α accumulation, as well as HIF-1α protein synthesis under hypoxic conditions in several cancer cell lines, including LNCaP prostate cancer cells, SkBr3 breast cancer cells, and CX-1 colon cancer cells, and suppressed HIF-1α protein synthesis in a concentration-dependent manner[120]. Interestingly, cycloheximide (an HIF-1 protein inhibitor) has the same effect on HIF-1α synthesis as quercetin [120]. In another study, Du et al. identified anticancer role of quercetin through the improvement of the therapeutic index of the anthracycline antibiotic doxorubicin (DOX) against 4T1 breast cancer cells. Quercetin suppressed intra-tumoral HIF-1αin 4T1 cells in a hypoxia-dependent manner. On the contrary, quercetin increased the accumulation of HIF-1α in healthy cells. Acquired data showed that quercetin promoted therapeutic index of DOX via its opposite effects on HIF-1α in healthy and cancer cells [121].

Epigallocatechin-3-gallate (EGCG) is a bioactive phenolic found in green tea with numerous health benefits, including possible anticancer activity [122]. In the PANC-1 pancreatic cancer cell line, EGCG suppressed proliferation and dose-dependently inhibited the expression of HIF-1α [123]. Cancer progression and angiogenic inhibition after treatment with EGCG were observed in HeLa cervical carcinoma and HepG2 hepatoma cells. EGCG significantly suppressed HIF-1α protein accumulation in these cells but did not affect HIF-1α mRNA expression. The mechanism underlying the HIF-1 inhibitive properties of EGCG is explained by its interference with the PI3K/Akt/mTOR pathway and translational mechanisms [124].

Deguelin, the rotenone flavonoid extracted from plants such as *Derris trifoliata* Lour. and *Mundulea sericea* (Willd.) A. Chev., modulates numerous signaling pathways [125]. Deguelin reduced the expression of HIF-1α in lung cancer (H1299), human squamous cell carcinoma (UMSCC38), prostate cancer (PC3), gastric cancer (MKN-45), and breast cancer (MCF-7) cell lines, as well as in vascular endothelial cells. In particular, deguelin inhibited *de novo* HIF-1α synthesis and promoted its proteasomal degradation [126]. Moreover, deguelin disrupted HSP90 function by binding to the ATP-binding pocket of chaperone, resulting in the proteasomal degradation of HIF-1α in H1299 xenografts [127]. Similarly, the inhibition of HSP90 activity by deguelin led to the suppression of HIF-1α in radioresistant lung cancer (H1299 and H226B) cells. Besides, athymic nude mice bearing H1299 and H226B xenografts were used to determine the inhibitory effects of deguelin on HSP90 stability. The combination of radiation and deguelin significantly decreased tumor growth and reduced HIF-1α expression in the analyzed xenografts [128].

Baicalein, a flavone isolated from *Scutellaria baicalensis* Georgi and its glucuronide baicalin, possess various beneficial properties for human health, including anticancer effects demonstrated in numerous studies [129,130,131,132]. Baicalein significantly reduced intracerebral tumor growth and proliferation and promoted apoptosis and cell cycle arrest in orthotopic U87 gliomas in mice. Interestingly, baicalein decreased the protein expression of HIF-1α in U87 gliomas [133]. Additionally, the suppression of HIF-1α by baicalein contributed to its reduction of cell viability in ovarian cancer (OVCAR-3 and CP-70) cell lines [134].

Chrysin is a flavone commonly occurring in propolis and honey [135,136]. Chrysin contributed to increased HIF-1α prolyl-hydroxylation, leading to its ubiquitination and subsequent degradation, and interfered with HSP90/HIF-1α connections, thus reducing HIF-1α stability in a human prostate cancer (DU145) cell line. Besides, chrysin modulated the expression of HIF-1α via the PI3K/Akt signaling pathway [137].

Common flavone luteolin suppressed HIF-1 activation within M2-like tumor-associated macrophages (TAMs) under hypoxic conditions [138].

Kaempferol, a prevalent flavonol obtained for example from tea, vegetables, and fruits, also exhibits anticancer efficacy [139]. Kaempferol demonstrated strong inhibitory effects on HIF-1 activity in Huh7 hepatocellular carcinoma cells through the mislocalization of HIF-1 into the cytoplasm due to p44/p42 MAPK inactivation; this decreased cell viability under hypoxic conditions [140]. Moreover, quercetin, baicalein, luteolin, and fisetin modulated HIF-1 transcriptional activity and its nuclear accumulation in HeLa cells. Flavonoids affected the hypoxia-response pathway, partially via stabilization of HIF-1α; on the other hand, these compounds decreased the transcriptional activity of HIF-1 in the same cancer cell line. Further analysis revealed that flavonoids affect the MAPK pathway and thus impair HIF-1α nuclear accumulation [141].

As mentioned above, flavonoids demonstrated strong anticancer efficacy via a decrease in HIF-activity. Interestingly, recent evidence suggested the anticancer activity of flavonoids also through the induction of HIF-1 activity. Quercetin inhibited proliferation via induction of HIF-1α expression and HIF-1 activity in HepG2 hepatoma cells under normoxia and hypoxia. Further analysis revealed that quercetin-mediated inhibition of proliferation is associated with the expression of p21WAF (cell cycle inhibitor) and knock-down of HIF-1α impairs these effects [142]. Similarly, quercetin induced HIF-1α and repressed cell proliferation by reducing the concentration of intracellular iron through chelation in HeLa cell line [143]. Furthermore, quercetin induced HIF-1α/2α accumulation in different human prostate adenocarcinoma cell lines. Quercetin was able to chelates irons that were necessary for HIF-1α/2α PHD activity and thus suppressed HIF-1α/2α ubiquitination [144]. Table 2 provides an overview of preclinical studies investigating the impact of flavonoids on HIF-1.

### 3.2. Flavonoids Targeting HIF-1 and Glucose Metabolism: Connections between Hypoxia and the Warburg Effect

Several studies identified the role of flavonoids in regulating the Warburg effect associated with HIF-1 [145]. In this regard, we emphasize the anticancer efficacy of flavonoids by regulating connections between HIF-1 and the components of glycolysis.

Apigenin is a flavone found in numerous vegetables and fruits [146]. As demonstrated in human pancreatic cell lines (S2-013 and CD18), apigenin reduced proliferation and angiogenesis and significantly suppressed the mRNA and protein expression of HIF-1α, VEGF, and GLUT1 under normoxic and hypoxic conditions [147].

Baicalein affected HIF-1α expression in glioma and ovarian cancer cells [133,134]. Moreover, baicalein increased the sensitivity of gastric cancer cells (AGS) to 5-fluorouracil (5-FU) under hypoxic conditions. Besides, baicalein suppressed the expression of glycolysis-associated enzymes including HKII, PDK1, and LDHA via inhibition of Akt-phosphorylation, which led to HIF-1α suppression [148].

Similarly, bavachinin, a prenylated flavanone, exerted antitumor effects by targeting HIF-1α in KB (HeLa derivatives) and osteosarcoma (HOS) cells. Moreover, its inhibitory effect on cellular metabolism was associated with the reduced transcription of genes, including GLUT1 and HKII. Bavachinin also significantly reduced tumor growth in vivo [149].

Epigallocatechin (EGC), a catechin derivative of *Spatholobus suberectus* Dunn or green tea, showed anticancer abilities by modulating glucose metabolism via HIF-1. EGC reduced LDHA activity in breast cancer (MCF-7 and MDA-MB-231) cell lines; notably, LDHA inhibition results from the dissociation of HSP90 from HIF-1α, resulting in HIF-1α proteasomal degradation. In vivo analysis identified the role of EGC in inhibiting tumor growth, suppressing LDHA and HIF-1α expression, and triggering apoptosis without detected adverse effects [150].

The prenylated isoflavones alpinumisoflavone (ALP) and 4′-O-methylalpinumisoflavone (4’-OM) showed anticancer effect by targeting HIF-1 in vitro. Both compounds inhibited HIF-1 activation in 4T1 breast cancer cells. Interestingly, 4’-OM inhibited the hypoxic induction of GLUT1 at the same concentration that inhibited HIF-1 activation. Further analysis suggested that HIF-1 inhibition occurred through the blocking of nuclear HIF-1α protein induction [151].

Naringin, a major flavanone of grapefruit and other citrus fruits, demonstrates several beneficial properties, including antioxidant and anticancer activities [152,153,154,155]. Naringin inhibited glucose metabolism in A375 melanoma cells in a concentration-dependent manner. The metabolic-regulatory properties of naringin are explained by the inhibition of PKM2, LDHA, and HIF-1α expression. Further studies revealed that naringin inhibited the Warburg phenotype in melanoma cells by reducing *c-Src* phosphorylation [156].

Oroxylin A, a flavone isolated from the same plant as baicalein (*S. baicalensis*), modulates glycolysis in cancer cells. Oroxylin A inhibited glycolysis-dependent proliferation in MDA-MB-231 cells via Sirtuin3-mediated destabilization and consequent HIF-1α destabilization. Therefore, HIF-1α destabilization led to the decreased expression of HKII and inhibition of glycolysis. In vivo analysis detected tumor growth inhibition associated with glycolysis suppression after oroxylin A intervention [157]. Wogonin is another constituent of *S. baicalensis* widely utilized in the treatment of numerous diseases [158]. The anticancer efficacy of wogonin was accompanied by decreases in HKII, PDK1, and LDHA expression, the suppression of lactate generation, and reduced glucose uptake in the HCT116 colon cancer cell line. Moreover, protein analysis revealed that wogonin could reduce HIF-1α expression by inhibiting the PI3K/Akt signaling pathway. Moreover, wogonin demonstrated metabolism-regulating activities through the downregulation of HIF-1α expression, suppression of glycolytic-related proteins, and inhibition of PI3K/Akt signaling in vivo [159].

An overview of flavonoids exerting regulatory effects on HIF-1 associated with glycolysis components is provided in Table 3.

### 3.3. Anticancer Effects of Flavonoids Mediated through Glucose Transporters and Enzymes of Glucose Metabolism

Glucose transporters (GLUTs) and enzymes of glucose metabolism, including HK, PK, LDHA, PFK, and PDK, are regulated by HIF-1 due to HRE’s presence. HIF-1 plays an essential role in the initiation of the expression of these enzymes. Therefore, it is appropriate to define the role of flavonoids in the regulation of signaling pathways associated with the hypoxia-glycolysis-cancer cascade [147,148,149,150,151,156,157,159,160]. As discussed below, the capacity of flavonoids to modulate enzymes of glucose metabolism and glucose transporters underscores their importance as anticancer agents that regulate HIF-1.

#### 3.3.1. Glucose Transporter (GLUT)

The dihydrochalcone phloretin, isolated from apple leaves, inhibited signals by type 2 glucose transporters (GLUT2) and thereby suppressed the migration and proliferation of MDA-MB-231 breast cancer cells [161]. Wogonin was found to upregulate p53 and p53-inducible glycolysis in colon cancer (HCT-116), ovarian cancer (A2780), and liver cancer (HepG2) cells and downregulated glucose transporter 1 (GLUT1) in cancer cells expressing wild type but not mutated p53. Wogonin also inhibited glycolysis in A2780 xenografts accompanied by the downregulation of GLUT1 [162]. Red wine and green tea flavonoids function as cis-allosteric activators of sugar uptake at low concentrations and as competitive inhibitors of GLUT1-mediated sugar uptake at higher concentrations, explaining their possible anticancer effectiveness [163]. A nanoliposomal encapsulation of celecoxib and genistein suppressed GLUT1 receptors; these effects reduced prostate cancer cell (PC-3, LNCaP) proliferation [164]. Similarly, a combinatorial liposomal formulation of plumbagin and genistein decreased the population of GLUT1 transporters in the same type of prostate cancer cells [165].

#### 3.3.2. Hexokinase II

In addition to GLUT, flavonoids also affect various enzyme systems, including HK. Luteolin-7-*O*-β-D-glucoside (LUT-7G), an HKII inhibitor, suppressed HKII and thereby repressed glycolytic pathway in the keratinocytes [166]. Quercetin inhibited glycolysis and proliferation of glycolysis-dependent hepatocellular carcinoma (SMMC-7721 and Bel-7402) cells by downregulating HKII; it also decreased HKII expression and restrained the growth of hepatocellular carcinoma xenografts in vivo [167]. Licochalcone A, a chalcone extracted from liquorice, suppressed HKII-mediated tumor glycolysis by downregulating Akt in gastric cancer (MKN45 and SGC7901) cells [168]. Furthermore, Tao et al. demonstrated that the synthetic flavonoid Gen-27 inhibited glycolysis and induced apoptosis of breast cancer (1H-I, MDA-MB-231, MCF-7, and MDA-MB-468) cells by suppressing HKII and consequently weakening interactions between HKII and voltage-dependent anion channels (VDAC) [169]. Similarly, the newly identified flavonoid GL-V9 downregulated HKII and dissociated it from mitochondrial VDAC, leading to the mitochondria-mediated apoptosis of breast cancer (MDA-MB-231, MCF-7) cells [170].

#### 3.3.3. Pyruvate Kinase

Targeting pyruvate kinase isoenzyme M2 (PKM2), which is considered the most important control point enzyme in glycolysis, is an important anticancer strategy [171]. PKM2 is overexpressed in various cancer types [171], and its expression is regulated by HIF-1α [172]. Wei et al. demonstrated low expression of pyruvate kinase isoenzyme M1 (PKM1) and high expression of PKM2 in liver cancer. Therefore, an increase in the PKM1/PKM2 ratio and the activation of hepatocyte nuclear factor 4 alpha (HNF-4α) to induce hepatoma differentiation and suppress cancer progression using oroxylin A could be therapeutically relevant in liver cancer [173]. Moreover, apigenin was suggested to be an allosteric inhibitor of PKM2 due to its ability to ensure a low PKM2/PKM1 ratio and restrain proliferation of colon cancer (HCT116) cells through a blockade of PKM2-dependent glycolysis [174].

#### 3.3.4. Lactate Dehydrogenase

The increased expression of lactate dehydrogenase (LDHA) in multiple cancers is associated with the promotion of glycolysis through the conversion of pyruvate into lactate [175]. Flavonoids modulate LDHA in various cancer models. Wogonin treatment suppressed LDHA activity in human gastric cancer (SGC-7901) and human lung adenocarcinoma (A549) cells [176]. Moreover, EGCG attenuated LDHA release in Sarcoma 180 tumor-bearing mice [177]. Furthermore, a synergistic evaluation of tangeretin-assisted platinum nanoparticles with doxorubicin revealed the capacity of such nanoparticles to increase LDHA leakage in osteosarcoma (U2OS) cells [178].

#### 3.3.5. Phosphofructokinase-1

Isoforms of phosphofructokinase-1 (PFK-1) are considered the pacemakers of glycolysis; therefore, they are highly expressed in various cancer types to support carcinogenesis via additional release of energy [179]. Phosphofructokinase platelet-type (PFKP) protein expression was positively associated with nodal expansion, estrogen receptor and progesterone receptor negativity, and overall reduced survival of breast cancer patients. Nevertheless, quercetin impaired the PFKP-LDHA signaling axis, and thus inhibited the migration of breast cancer (MDA-MB-231) cells induced by aerobic glycolysis [180]. Moreover, EGCG inhibited the expression and activity of PFK in hepatocellular carcinoma (HCC-LM3 and HepG2) cells [179]. Similarly, EGCG enhanced the effects of gemcitabine and further suppressed PFK and PK levels in pancreatic cancer (Panc-1, MIA PaCa-2) cells [181].

#### 3.3.6. Pyruvate Dehydrogenase Kinase

The potential importance of pyruvate dehydrogenase kinase 3 (PDK3) in metabolic cancer switching supports the idea of its role as a cancer therapy target. The overexpression of PDK3 correlates with cancer progression. However, quercetin interacts with the crucial residues of the active site cavity of PDK3 and exerts conformational fitting. In addition, quercetin inhibited PDK3 in hepatocellular carcinoma (HepG2) and lung cancer (A549) cells [182].

**Figure 4:**
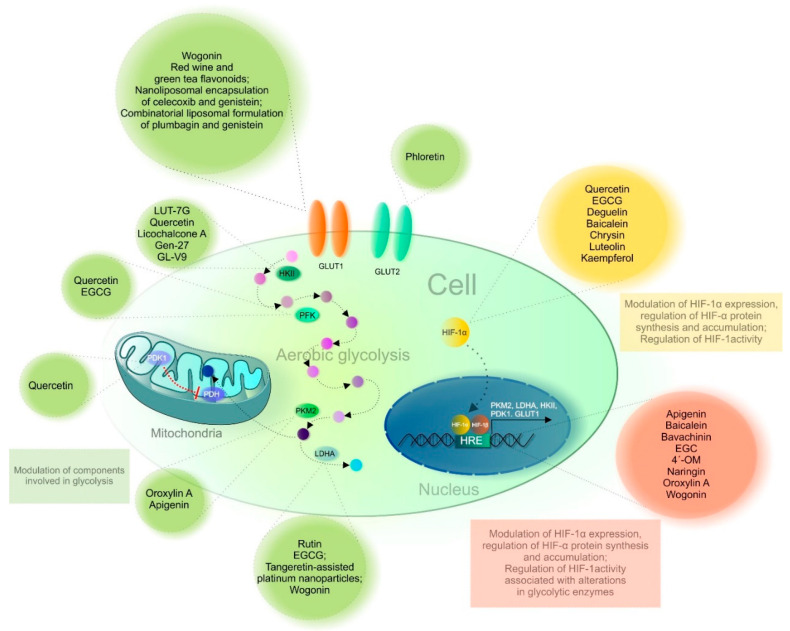
Flavonoids targeting HIF-1 and critical components of glycolysis. Abbreviations: EGCG, epigallocatechin-3-gallate; EGC, epigallocatechin; 4′-OM, 4′-O methylalpinumisoflavone; LUT-7G, Luteolin-7-*O*-β-D-glucoside; *HK 2*, *hexokinase 2*; *PFK*, phosphofructokinase; PKM 2, pyruvate kinase muscle isoform *2*; *LDHA*, *lactate dehydrogenase A*; PDH, pyruvate dehydrogenase; PDK1, pyruvate dehydrogenase kinase 1; HRE, hypoxia-response elements; HIF-1α/1β, hypoxia-inducible factor 1α/1β; GLUT1/2, glucose transporter 1/2.

Indeed, flavonoids exert a wide range of biological effects. Based on the discussed preclinical research results (Table 4), we can highlight the essential anticancer capacity of flavonoids by modulating HIF-1-regulated glucose transporters and enzymes of glucose metabolism in tumor cells. Figure 4 presents an overview of flavonoids used as repressors of the Warburg phenotype via regulation of HIF-1 as well as critical components of glycolysis.

## 4. Conclusions

HIF-1 is one of the critical players in the reprogramming of cancer cell metabolism. Its effects include regulating the expression of genes that encode glucose transporters, glycolytic enzymes, and pyruvate dehydrogenase kinase 1; by this mechanism, HIF-1 could directly trigger the Warburg effect. Targeting HIF-1α and hypoxia-related effector molecules could impair cancer cell survival by attenuating glucose metabolic processes or inhibiting VEGF-induced angiogenesis and survival-promoting signaling pathways. The inhibition of the HIF-1-dependent Warburg effect is becoming a hot topic in cancer research. Flavonoids have a wide range of biological activities and seem to be effective chemopreventive and therapy-sensitizing cancer agents. Preclinical research demonstrated that flavonoids could suppress tumor hypoxia-induced glycolytic processes and consequently suppress the cellular metabolism and/or increase susceptibility of cancer cells to radio- and chemotherapy. In addition, effects of flavonoids on glucose metabolism and cellular energy production contribute to their broader implications for apoptosis induction, inhibition of metastasis, and reduction of cell viability in cancer cells. The emerging interplay between altered gene expression and metabolism in cancer cells suggests that many of the oncostatic effects described in flavonoids prevent the deregulation of cancer cell metabolism, and growing evidence from scientific databases confirms this hypothesis. Although there are currently no clinical studies focused on the correlation of HIF-1 and flavonoids in cancer patients, preclinical data indicate that flavonoids may be useful in cancer management. However, several complications associated with the bioavailability and safety of naturally occurring phenols limit their application in the clinical sphere. Most flavonoids are safe, but eventual side effects are associated with an excessive intake resulting in hemolytic anemia or mild gastrointestinal symptoms. Moreover, excessive metabolization of flavonoids in the intestine, colon, liver, as well as the participation of gut microbiota also limit their bioavailability and applicability. Only more-in depth investigations on flavonoids bioavailability and safety can improve their biological activity and eliminate side effects. Despite the above-mentioned complications, flavonoids, as supposed modulators of cancer cell metabolism, might represent clinically viable chemo-adjuvants in both chemoprevention and treatment settings; however, further comprehensive and controlled investigations are warranted.

## Figures and Tables

**Table 1:** An overview of HIF-1 inhibitors.

Agents.	Effect on HIF-1	References
EZN-2968	Regulation of HIF-1α mRNA expression	[85]
Topotecan	Regulation of HIF-1α mRNA expression	[86,87]
2-Methoxyestradiol	Modulation of HIF-1α and HIF-2α protein synthesis	[88]
KC7F2	Modulation of HIF-1α protein synthesis	[89,90]
Geldanamycin, tanespimycin, and alvespimycin	Disruption of HIF-1α protein stabilization and accumulation	[91,92,93]
Vorinostat (HDACi)	Disruption of HIF-1α protein stabilization and accumulation	[94]
Cyclo-CLLFVY	Inhibition of HIF-1 α heterodimerization	[19,95]
Acriflavine	Inhibition of HIF-1α heterodimerization	[96]
Doxorubicin, daunorubicin	Inhibition of binding of HIF-1 to its target gene sequences	[97]
Chetomin	Inhibition of the HIF-1 transcriptional activity by targeting HIF-1α/p300	[98]

Abbreviations: HIF-1, hypoxia-inducible factor 1; HIF-1α/2α, hypoxia-inducible factor 1α/2α; HDCAi, histone deacetylase inhibitor.

**Table 2:** Role of flavonoids in the modulation of HIF-1.

Flavonoid	Study Details	Mechanism of Action	Effect on HIF-1	Reference
Quercetin	Colon cancer cells (HCT116), BALB nu/nu mice	Inhibition of AMPK. Probability of cross-connection between suppression of AMPK and decrease in HIF-1 activity	Decrease in HIF-1 activity	[119]
	Prostate cancer cells (LNCaP), breast cancer cells (SkBr3), and colon cancer cells (CX-1)	Modulation of the balance between HIF-1α translation and degradation	Inhibition of HIF-1α protein synthesis and accumulation	[120]
	Murine mammary carcinoma (4T1)	Promotion of HIF-1α degradation in cells.	Suppression of intra-tumoral HIF-1α	[121]
EGCG	Pancreatic cancer cells (PANC-1)	Promotion of HIF-1α protein degradation and/or interaction with HIF-1α protein translational pathway	Decrease in HIF-1α protein expression	[123]
	Human liver cancer cells (HepG2) and cervical cancer cells (HeLa)	Inhibition of HIF-1α expression by interfering with PI3K/Akt/mTOR pathway and translational apparatus of cancer cells	Inhibition of HIF-1α protein accumulation	[124]
Deguelin	Lung cancer cells (H1299), human squamous cell carcinoma (UMSCC38), prostate cancer cells (PC3), gastric cancer cells (MKN-45), breast cancer cells (MCF-7), and vascular endothelial cells	Suppression of HIF-1α expression by the inhibition of protein synthesis, and its degradation through the ubiquitin- and proteasomal-dependent manner	Reduction of HIF-1α expression	[126]
	Lung cancer (H1299) xenografts	Interaction with the ATP-binding pocket of Hsp90 and disruption of its function resulting in HIF-1α degradation via ubiquitin-mediated manner	Degradation of HIF-1α	[127]
	Lung cancer cells (H1299 and H226B); athymic nude mice bearing H1299 and H226B cells	Suppression of interaction between Hsp90 and HIF-1α	Suppression of HIF-1α expression	[128]
Baicalein	Mice with human glioblastoma cells (U87)	Suppression of HIF-1α/VEGF pathway	Decrease in HIF-1α protein expression	[133]
	Ovarian cancer cells (OVCAR-3a and CP-70)	Inhibition of HIF-1α expression by baicalein at concentration 20-μM and 40-μM.	Suppression of HIF-1α expression	[134]
Chrysin	Prostate cancer cells (DU145)	Enhancing HIF-1α degradation via promotion of the prolyl hydroxylation of HIF-1α ODD, resulting in proteasomal-mediated degradation of HIF-1α. Regulation of HIF-1α expression via PI3K/Akt pathway	Inhibition of HIF-1α expression and protein stability	[137]
Luteolin	The mouse macrophage cell line RAW264.7	Regulation of HIF-1α-VEGF/MMP9 signaling pathway	Suppression of HIF-1 activation	[138]
Kaempferol	hepatocellular cancer cells (Huh7)	HIF-1α mislocalization into the cytoplasm due to p44/42 MAPK inactivation, resulting in the suppression of HIF-1 activity	Decrease in HIF-1 activity	[140]
Quercetin, baicalein, luteolin, and fisetin	cervical cancer cells (HeLa)	Flavonoids affect HIF-1 transcriptional activity via impairing the MAPK pathway resulting in inhibition of phosphorylation and nuclear accumulation of HIF-1α	Inhibition of HIF-1α protein accumulation and HIF-1 transcriptional activity	[141]
Quercetin	Human hepatoma cells (HepG2)	Quercetin prolongs HIF-1α protein half time. Knock-down of the HIF-1α disrupts quercetin-mediated inhibition of cell proliferation	Induction of the HIF-1α expression and HIF-1 activity	[142]
	cervical cancer cells (HeLa)	Quercetin induces HIF-1α and inhibits cell proliferation via iron chelation	Induction of the HIF-1α expression	[143]
	Human prostate adenocarcinoma cells (LNCaP, DU-145 and PC-3)	Quercetin containing iron-chelating moieties chelates cellular irons that are cofactors of HIF-1α/2α PHD leading to HIF-1a accumulation	Induction of HIF-1α/2α accumulation	[144]

Abbreviations: EGCG, epigallocatechin gallate; HIF-1, hypoxia-inducible factor 1; HIF-1α, hypoxia-inducible factor 1α.

**Table 3:** Flavonoids targeting HIF-1-associated components contributing to the Warburg effect.

Flavonoids	Study Details	Mechanism of Action	Effect on HIF-1/Glycolysis Components	Reference
Apigenin	Human pancreatic cancer cells (S2-013 and CD18)	Downregulation of HIF-1α and GLUT-1 mRNA expression. Repression of any hypoxia-mediated induction of GLUT-1 expression. Significant reduction of the HIF-1 protein level	Inhibition of HIF-1α and GLUT1	[147]
Baicalein	Human gastric cancer cells (AGS)	Inhibition of glycolysis through the regulation of PTEN/Akt/HIF-1α signaling pathway	Suppression of HIF-1α expression; suppression of HKII, PDK1, LDHA	[148]
Bavachinin	HeLa derivatives (KB), Human osteosarcoma cells (HOS), KB xenografts	Promotion of VHL-HIF-1α interaction as a consequence of the elevated PHD2 activity. A decrease in glucose metabolism and energy level modulated by hypoxia due to bavachinin intervention	Inhibition of HIF-1 activity; decreased transcription of HKII and GLUT1	[149]
EGC	Breast cancer cells (MCF-7 and MDA-MB-231) and MCF-7, MDA-MB-231 xenografts	Promotion of HIF-1α proteasomal-mediated degradation via Hsp90. EGC modulates the interaction of Hsp90/HIF-1α. Acceleration in HIF-1α proteasomal degradation correlates with regulation of LDHA	Inhibition of LDHA activity; induction of HIF-1α proteasomal degradation; decrease in HIF-1α/LDHA expression *in vivo*	[150]
ALP, 4’-OM	Breast cancer cells (T47D)	Inhibition of hypoxia-induced HIF-1 activation by ALP and 4’-OM. 4’-OM inhibits HIF-1 activation via suppression of mitochondrial electron transport chain and inhibition of protein synthesis	Inhibition of HIF-1 activity; inhibition of hypoxic induction of GLUT1	[151]
Naringin	Human melanoma cells (A375)	Significant anticancer impact of naringin on HIF-1α, PKM2, LDHA expression mediated by suppression of phosphorylation of Tyr418 of c-Src	Inhibition of PKM2, LDHA, and HIF-1α	[156]
Oroxylin A	Breast cancer cells (MDA-MB-231)	Downregulation of HIF-1α via increasing PHD activity mediated by SIRT3. Glycolysis was inhibited by suppression of HIF-1 activity	HIF-1α destabilization; suppression of HKII expression	[157]
Wogonin	Colon cancer cells (HTC116) and Balb/C mice	Inhibition of HIF-1α and glycolysis-related proteins was mediated by suppression of PI3K/Akt signaling pathway leading to downregulation of PI3K/Akt-dependent transcriptional activity	Suppression of HIF-1α expression; decreases in HKII, PDK1, and LDHA expression	[159]

Abbreviations: EGC, epigallocatechin; ALP, alpinumisoflavone; 4’-OM, 4’-O-methylalpinumisoflavone; HKII, hexokinase 2; PDK1, pyruvate dehydrogenase kinase 1; LDHA, lactate dehydrogenase; GLUT-1, glucose transporter 1; HIF-1, hypoxia-inducible factor 1; HIF-1α, hypoxia-inducible factor 1α; Hsp90, Heat shock protein 90.

**Table 4:** Effects of flavonoids on glucose transporters and enzymes of glucose metabolism.

Enzyme	Flavonoid	Study Details	Mechanism of Action	Effects	Reference
Glucose transporters					
GLUT2	Phloretin	Breast cancer cells (MDA-MB-231)	Inhibition of GLUT2 → accumulation of MDA-MB231 cells in the G0/G1 phase	suppression of migration and proliferation	[161]
GLUT1	Wogonin	Colon cancer (HCT-116), ovarian cancer (A2780), and liver cancer (HepG2) cells, A2780 xenografts	Suppression of glucose metabolism followed by upregulated p53 mRNA and protein level (wt-p53 cancer cells) and regulation of p53 downstream glycolytic factors	Upregulated p53 and p53-inducible glycolysis in cancer cells and decreased GLUT1 in cells expressing wild type, but not mutated p53. Inhibition of glycolysis was accompanied by the downregulation of GLUT1 in xenografts	[162]
	Red wine and green tea flavonoids	The evaluation of structure–function relationships in flavonoid–GLUT1 interactions	Stimulation of GLUT1-mediated sugar uptake at low concentrations → transport inhibition as the concentration raises (suggesting that at least two flavonoid-binding sites modulate GLUT1 function)	Act as: cis-allosteric activators of sugar uptake at low concentrations; and competitive inhibitors of GLUT1-mediated sugar uptake at higher concentrations	[163]
	Nanoliposomal encapsulation of celecoxib and genistein	Prostate cancer cells (PC-3, LNCaP)	Key processes behind the inhibition of prostate cancer cells: enhanced reactive oxygen species, decreased cellular GSH concentration, inhibited COX-2 synthesis and Glut-1 receptors	Suppressed GLUT1 receptors → prevention of prostate cancer cell proliferation	[164]
	Combinatorial liposomal formulation of plumbagin and genistein		Genistein (Glut-1 transporter protein inhibitor) induces high reactive oxygen species generation associated with AMPK signaling pathway. Low uptake of glucose → decreased metabolism of prostate cancer cells and simultaneous generation of reactive oxygen species and low GSH concentration → cell death	Decreased population of GLUT1 transporters	[165]
**Enzymes of glucose metabolism**					
HKII	LUT-7G	Keratinocytes	LUT-7G suggested to represent a strong HKII inhibitor via the binding in the active sites	HKII suppression → repression of the glycolytic pathway	[166]
	Quercetin	Hepatocellular carcinoma cells (SMMC-7721 and Bel-7402) and murine xenograft model	Quercetin suppresses glycolysis through Akt-mTOR pathway-mediated HKII regulation	Inhibition of glycolysis and proliferation of glycolysis-addicted HCC cells (by reduced HKII) and decrease of HKII expression *in vivo*	[167]
	Licochalcone A	Gastric cancer cells (MKN45 and SGC7901)	Licochalcone A inhibits glycolysis mainly through the blockade of Akt signaling pathway	Suppression of HKII-mediated tumor glycolysis	[168]
	Gen-27	Breast cancer cells (1H-I, MDA-MB-231, MCF-7 and MDA-MB-468)	The potential of Gen-27 to inhibit glycolysis and displaced HKII from mitochondrial membrane to the cytosol → blockage of its preferential access to ATP for glucose phosphorylation or preventing mechanism of cancer growth and immortality	Inhibition of glycolysis and induction of apoptosis (through HKII suppression accompanied by weakened interactions of HKII and VDAC)	[169]
	GL-V9	Breast cancer cells (MDA-MB-231, MCF-7)	GL-V9 disrupts GSK-3β-modulated mitochondrial binding of HKII	Downregulation of HKII and disruption of mitochondrial binding of HKII resulting in apoptosis	[170]
PKM1, PKM2	Oroxylin A	Liver cancer model	Oroxylin A enhanced the protein expression of HNF-4α and its binding to the promoter region of HNF-1α and promoted direct interaction between PKM1 and HNF-4α in the nucleus	Increased PKM1/PKM2 ratio → HNF-4α activation → induction of hepatoma differentiation and suppression of cancer progression	[173]
	Apigenin	Colon cancer cells (HCT116)	The potential of apigenin to ensure a low PKM2/PKM1 ratio through blockage of the β-catenin/c-Myc/PTBP1 signal pathway	Apigenin → allosteric PKM2 inhibitor (can ensure a low PKM2/PKM1 ratio and restrain the proliferation of colon cancer cells through a blockade of PKM2-dependent glycolysis)	[174]
LDHA	Wogonin	Human gastric cancer cells (SGC-7901) and human lung adenocarcinoma cells (A549)	Effects of wogonin on energy metabolism: affecting ATP generation and the activities of energy associated with metabolism	Reduced LDHA activity	[176]
	EGCG	Evaluation of effects of EGCG on doxorubicin-induced cardiotoxicity in Sarcoma 180 tumor bearing mice	EGCG-exerted heart benefits related to reduced LDH release	Attenuation of LDHA release.	[177]
	Tangeretin-assisted platinum nanoparticles	Osteosarcoma cells (U2OS)	Tangeretin-assisted platinum nanoparticles promote LDHA leakage	Increase of LDHA leakage and cell death	[178]
PFK	Quercetin	Breast cancer cells (MDA-MB-231)	The ability of quercetin to impair PFKP-LDHA signaling → inhibiting migration of cancer cells mediated by aerobic glycolysis	Impairment of the PFKP-LDHA signaling axis → inhibition of cell migration induced by aerobic glycolysis	[180]
	EGCG	Hepatocellular carcinoma cells (HCC-LM3 and HepG2)	EGCG inhibits glycolysis (especially PFK activity) in aerobic glycolytic HCC cell lines	Inhibition of PFK expression and activity	[179]
		Pancreatic cancer cells (Panc-1 and MIA PaCa-2)	EGCG inhibits glycolysis through repressing rate-limiting enzymes (PFK and PKM2)	Suppression of PFKP and PKM2 levels	[181]
PDK	Quercetin	Hepatocellular carcinoma cells (HepG2) and liver cancer (A549) cells	Quercetin binds with PDK3 and significantly inhibits its kinase activity	Interaction with residues of the active site cavity of PDK3 (conformational fitting). PDK3 inhibitory potential in cancer cells	[182]

Abbreviations: COX-2, cyclooxygenase; EGCG, epigallocatechin-3-gallate; GLUT, glucose transporters; GLUT1, glucose transporter type 1; GLUT2, glucose transporter type 2; GSH, glutathione; HEKII, hexokinase II; HK, hexokinases; HNF-4α, hepatocyte nuclear factor 4 alpha; LDHA, lactate dehydrogenase; LUT-7G, luteolin-7-*O*-β-D-glucoside; PDK, pyruvate dehydrogenase kinase; PDK3, pyruvate dehydrogenase kinase 3; PFK, phosphofructokinase; PFK-1, phosphofructokinase-1; PFKP, phosphofructokinase platelet-type; PK, pyruvate kinase; PKM1, pyruvate kinase isoenzyme M1; PKM2, pyruvate kinase isoenzyme M2; PTBP1, polypyrimidine tract binding protein; VDAC, voltage-dependent anion channel.
